# The Early External Cephalic Version (ECV) 2 Trial: an international multicentre randomised controlled trial of timing of ECV for breech pregnancies

**DOI:** 10.1111/j.1471-0528.2010.02837.x

**Published:** 2011-02-04

**Authors:** EK Hutton, ME Hannah, SJ Ross, M-F Delisle, GD Carson, R Windrim, A Ohlsson, AR Willan, A Gafni, G Sylvestre, R Natale, Y Barrett, JK Pollard, MS Dunn, P Turtle, for the Early ECV2 Trial Collaborative Group

**Affiliations:** a Department of Obstetrics and Gynecology (Midwifery), McMaster University Faculty of Health SciencesHamilton, ON; b Department of Obstetrics and Gynaecology, Sunnybrook Health Sciences Centre, University of TorontoToronto, ON; c Department of Obstetrics and Gynaecology, University of CalgaryCalgary, AB; d Department of Obstetrics and Gynaecology, BC Women's Hospital, University of British ColumbiaVancouver, BC; e Department of Obstetrics and Gynecology, Regina Qu'Appelle Health RegionRegina, SK; f Mount Sinai Hospital, Departments of Obstetrics and Gynaecology and Health Policy, Management and Evaluation, University of TorontoToronto, ON; g Mount Sinai Hospital, Departments of Paediatrics, Obstetrics and Gynaecology and Health Policy, Management and EvaluationToronto, ON; h Program in Child Health Evaluative Sciences, Sick Kids Research Institute, Department of Public Health Sciences, University of TorontoToronto, ON; i Centre for Health Economics and Policy Analysis, Department of Clinical Epidemiology and Biostatistics, McMaster UniversityHamilton, ON, Canada; j Weill Cornell Medical CollegeNew York City, NY, USA; k Department of Obstetrics and Gynecology and Pediatrics, Schulich School of Medicine, University of Western OntarioLondon, ON, Canada; l Department of Newborn and Developmental Paediatrics, Sunnybrook Health Sciences Centre, University of TorontoToronto, ON, Canada; m EECV2 Trial Consumer RepresentativeToronto, ON Canada

**Keywords:** Breech pregnancy, caesarean section, external cephalic version, fetal version, randomised controlled trial

## Abstract

**Objective:**

To investigate whether initiating external cephalic version (ECV) earlier in pregnancy might increase the rate of successful ECV procedures, and be more effective in decreasing the rate of non-cephalic presentation at birth and of caesarean section.

**Design:**

An unblinded multicentred randomised controlled trial.

**Setting:**

A total of 1543 women were randomised from 68 centres in 21 countries.

**Population:**

Women with a singleton breech fetus at a gestational age of 33^0/7^ weeks (231 days) to 35^6/7^ weeks (251 days) of gestation were included.

**Methods:**

Participants were randomly assigned to having a first ECV procedure between the gestational ages of 34^0/7^ (238 days) and 35^6/7^ weeks of gestation (early ECV group) or at or after 37^0/7^ (259 days) weeks of gestation (delayed ECV group).

**Main outcome measures:**

The primary outcome was the rate of caesarean section; the secondary outcome was the rate of preterm birth.

**Results:**

Fewer fetuses were in a non-cephalic presentation at birth in the early ECV group (314/765 [41.1%] versus 377/768 [49.1%] in the delayed ECV group; relative risk [RR] 0.84, 95% CI 0.75, 0.94, *P* = 0.002). There were no differences in rates of caesarean section (398/765 [52.0%] versus 430/768 [56.0%]; RR 0.93, 95% CI 0.85, 1.02, *P* = 0.12) or in risk of preterm birth (50/765 [6.5%] versus 34/768 [4.4%]; RR 1.48, 95% CI 0.97, 2.26, *P* = 0.07) between groups.

**Conclusion:**

External cephalic version at 34–35 weeks versus 37 or more weeks of gestation increases the likelihood of cephalic presentation at birth but does not reduce the rate of caesarean section and may increase the rate of preterm birth.

## Introduction

The fetus presents as a breech in 3–4% of all full-term singleton pregnancies and many of these pregnancies are delivered by caesarean section.^1^,^2^ External cephalic version (ECV) is an obstetrical procedure used during pregnancy to try to turn a breech fetus to cephalic by externally manoeuvring the fetus through the maternal abdomen. A Cochrane review reported that ECV at full-term gestation (≥37 weeks) decreases both the likelihood that the fetus will be in a non-cephalic presentation at birth and the need for caesarean section, and concluded that ECV should be recommended for all women with a breech fetus at term when there is no contraindication.^3^–^5^ However, ECV is unsuccessful in about 40% of attempts.^6^,^7^

We hypothesised that initiating ECV earlier in the pregnancy (before the fetal breech descends into the pelvis and while the maximum amount of amniotic fluid is present) might increase the rate of successful ECV procedures, and decrease both the rates of non-cephalic presentation at birth and of caesarean section. We undertook a pilot trial to determine if beginning ECV somewhat earlier than term (34–35 weeks) might be more effective than beginning it at term (37–38 weeks) in terms of decreasing the rate of non-cephalic presentation at birth.^8^ The pilot study reported a rate of non-cephalic presentation at birth in the early ECV group of 66/116 (56.9%) compared with 77/116 (66.4%) in the delayed ECV group (relative risk [RR] 0.86, 95% CI 0.70, 1.05, *P* = 0.09). Although the difference was not statistically significant, we felt that the results were sufficiently promising to justify a larger trial. A Cochrane Systematic Review confirmed this finding and recommended that further trials be conducted.^9^ The Early ECV 2 Trial was undertaken to answer the primary research question, ‘For women with a fetus in breech presentation, does early ECV (at 34^0/7^ weeks to 35^6/7^ weeks of gestation) versus delayed ECV (not before 37^0/7^ weeks of gestation) decrease or increase the likelihood of caesarean section?’ and the secondary research question, ‘Is the risk of preterm birth (<37^0/7^ weeks of gestation) higher or lower with early versus delayed ECV?’.

## Methods

The study was funded by the Canadian Institutes of Health Research (CIHR) and was co-ordinated jointly at Sunnybrook and Women's College Health Sciences Centre in Toronto, the University of British Columbia in Vancouver and McMaster University in Hamilton, Canada.

A pragmatic, multicentred, parallel group randomised controlled trial design, with prognostic stratification for parity (0 and ≥1) and centre, was used to test for superiority. Individual women were randomised using computer-generated random block sizes and 1:1 allocation. Randomisation was centrally controlled with a computerised randomisation program accessible by a toll-free 24-hour, 7-day-a-week telephone service. Baseline data were collected before randomisation. Data were collected on carbonless duplicate paper forms and the original copy was mailed to the Co-ordinating Centre where it was scanned into a TELEform™ data management system (Autonomy Cardiff Software, Vista, CA, USA). Logic and range checks were used to verify the accuracy of the data.

The study received ethical approval at the co-ordinating sites and all participating centres; participating women gave consent before randomisation. Pregnant women with a singleton fetus in a breech presentation who had a recent screening ultrasound and were between 33^0/7^ weeks (231 days) and 35^6/7^ weeks (251 days) of gestation were eligible for the study. Women were ineligible when they presented with contraindications to ECV (such as fetal heart rate abnormalities, placental abruption, major life-threatening fetal anomalies, uterine anomalies, hyper-extended fetal head, rupture of fetal membranes, severe oligohydramnios or hydramnios); contraindications to early ECV (such as increased risk of preterm labour or placental abruption); or contraindications to labour or vaginal birth (such as placenta praevia, previous classical caesarean section); or if they had been prior participants in the trial; were at increased risk of unstable lie (such as grand multiparity); or if they planned to give birth by caesarean section even if the fetus turned to a cephalic position, or if they planned a vaginal birth if the fetus remained breech.

### Settings

Centres were invited to participate in the trial if they had clinicians who were experienced in ECV and birth facilities that were deemed to meet Canadian standards as detailed in Table 1. The ECV procedures were undertaken or supervised by experienced clinicians. Experienced clinicians were those who judged themselves to be skilled and experienced in the ECV procedure and who's Heads of Departments agreed with that judgement. This definition of experience has been demonstrated to be robust.^10^

**Table 1:** Requirements for participating centres and outcome definitions used in EECV2 Trial

	Definition
Requirements for participating centres	Participating centres must be able to:
	•ensure that an experienced clinician will undertake all ECV procedures
	•have the anaesthetic, obstetrical and nursing staff to be able to undertake a caesarean section, if necessary,usually within 30 minutes of making the decision to do so
	•provide suitable facilities and qualified neonatal staff who are able to resuscitate a baby with respiratorydepression by giving oxygen (by mask, bag and mask or ventilator)
	•provide ventilation by endotracheal intubation and positive pressure ventilation
	•give intravenous therapy and blood transfusion and use surfactant;
	•obtain a neonatal head ultrasound, if necessary.
Perinatal or neonatal mortality or serious neonatal morbidity	One or more of:
	•death (stillbirth or neonatal death 0–27 days after birth, excluding lethal anomalies)
	•birth trauma (spinal cord injury, basal skull fracture or depressed skull fracture, long bone fracture, peripheralnerve injury, subdural or intracerebral haemorrhage)
	•Apgar score of <4 at 5 minutes
	•abnormal level of consciousness (coma, stupor or decreased response to pain)
	•neonatal seizures before 72 hours of age
	•need for assisted ventilation for ≥24 hours via endotracheal tube initiated within 72 hours after birth
	•infection (septicaemia [positive blood culture] or meningitis [positive cerebrospinal fluid culture]) determinedwithin 72 hours of birth
	•necrotising enterocolitis
	•bronchopulmonary dysplasia
	•Grade III or IV intraventricular haemorrhage
	•cystic periventricular leucomalacia.
Serious fetal complications	One or more of:
	•preterm prelabour rupture of the membranes
	•placental abruption requiring obstetrical intervention to effect birth
	•preterm labour followed by preterm birth
	•abnormalities of the fetal heart rate before labour requiring obstetrical intervention to effect birth.
Maternal mortality or serious maternal morbidity	One or more of the following during pregnancy, labour, birth or within the first 28 days postpartum:
	•maternal death
	•haemorrhage (documented blood loss of >1500 mL, blood transfusion required, or need for dilation/curettage ormanual removal of the placenta after delivery),
	•laparotomy excluding caesarean section or tubal ligation
	•genital tract injury (hysterectomy, vulvar or perineal haematoma requiring evacuation, symptomatic broadligament haematoma confirmed by ultrasound, CT or MRI, intraoperative damage to bladder, ureter or bowelrequiring repair, fistula involving the genital tract, or third or fourth degree perineal tear involving the analsphincter and/ or mucosa)
	•thromboembolism (deep vein thrombosis, thrombophlebitis, or pulmonary embolism) requiring anticoagulanttherapy
	•systemic infection (temperature of 38.5°C or more on two or more occasions at least 24 hours apart notincluding the first 24 hours, or pneumonia [confirmed by X-ray], or sepsis [confirmed by blood culture]),
	•major medical life-threatening illness (such as adult respiratory distress syndrome, amniotic fluid embolism,disseminated intravascular coagulation, bowel obstruction, or paralytic ileus [requiring nasogastric suctioning])
	•wound infection (requiring prolongation of hospital stay, readmission to hospital or repeated treatment as anoutpatient), dehiscence, or breakdown
	•other serious maternal complication.

### The intervention

**Figure 1:**
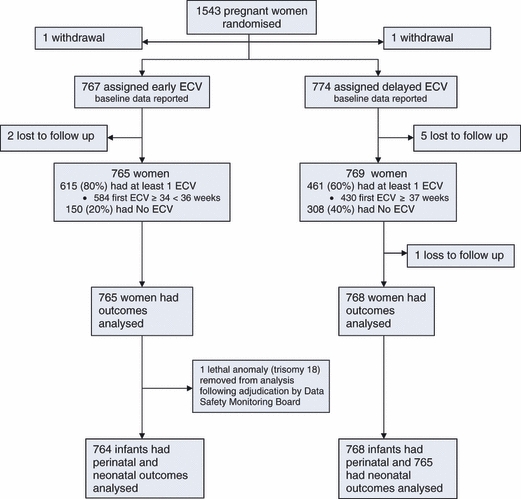
Trial profile.

A screening ultrasound was undertaken at 32^0/7^–35^6/7^ weeks of gestation (224–251 days) and within 1 week of randomisation to confirm breech presentation and rule out contraindications to ECV (see Figure 1). To allow time for booking of procedures, women were randomised at a gestational age as early as 33^0/7^ weeks and up to 35^6/7^ weeks of gestation, but no ECV was to be undertaken before 34^0/7^ weeks of gestation. The nature of the intervention did not lend itself to blinding of either participants or clinicians. The first ECV procedure was to be performed in the early ECV group between 34^0/7^ and 35^6/7^ weeks of gestation and within 7 days following randomisation and in the delayed ECV group at or after 37^0/7^ weeks of gestation. All attempts undertaken to manoeuvre the fetus at one visit were considered part of one procedure. In either group, if a procedure was unsuccessful, or if a fetus later reverted to non-cephalic, a repeat ECV procedure could be performed at a later date at the discretion of the care provider in consultation with the woman.

Immediately before the ECV procedure, women were reassessed to ensure eligibility for ECV including confirmation of fetal presentation by ultrasound. Fetal wellbeing was assessed by continuous fetal heart rate monitoring for 20 minutes to ensure a normal baseline rate, good variability and no evidence of decelerations, and was then monitored intermittently during the ECV procedure using auscultation, Doppler, or ultrasound viewing of fetal heart rate. The protocol recommended the use of tocolytic agents to relax the uterus during the ECV but their use was left to the discretion of the healthcare provider, as was the decision to use regional analgesia to facilitate ECV. Clinicians were directed to use the same approach to tocolytics and to regional analgesia for women in both arms of the trial.

The ECV procedure was discontinued if fetal heart tones were non-reassuring, if it was not easily accomplished, or if the woman reported undue discomfort. Fetal presentation was confirmed by ultrasound immediately following all procedures. Trial participants were monitored for at least 30 minutes following procedures to confirm fetal movement on ultrasound and a reactive fetal heart rate on continuous fetal heart rate monitoring. Anti-D immunoglobulin was recommended for all rhesus-negative women following the procedure.

Caesarean section was recommended to all women with a fetus that remained non-cephalic at the time of birth including in situations where labour began spontaneously.^11^,^12^ Vaginal birth was planned for all fetuses who were cephalic at onset of labour when there was no contraindication. All other aspects of care during pregnancy, labour and birth were determined by the woman and her caregiver.

Compliance with the timing of the ECV procedures in the two groups was assessed quarterly, for the trial as a whole and by centre, and reasons for non-compliance were reviewed with centres as needed. Site visits were made during study promotional visits and included random chart audits of selected data fields as well as a review of the study facility using an evaluation check list based on the Guidelines for Good Clinical Research Practice.^13^

To avoid bias, the gestational age determined before randomisation was used in determining gestational age at birth and the rate of preterm birth. Members of the independent Data Safety and Monitoring Board reviewed all stillbirths and neonatal deaths, blinded to allocation group, for the existence of any anomaly considered incompatible with life and to make a determination regarding exclusion of any women from the analysis of perinatal/neonatal outcomes. Women and infants were followed until 28 days after birth or until hospital discharge, whichever was later.

### Outcome measures

The primary outcome was the rate of caesarean section. The secondary outcome was the rate of preterm birth before 37 weeks of gestation. Other outcomes included: non-cephalic presentation at birth; admission to the neonatal intensive care unit for ≥24 hours; perinatal or neonatal mortality or serious neonatal morbidity; serious fetal complications; maternal death or serious maternal morbidity; pain experienced during the procedure; and maternal satisfaction. Women provided other information regarding their likes and dislikes about ECV and about trial participation and these results will be reported elsewhere. A health economic analysis is planned. Definitions are provided in Table 1.

Pain experienced during the procedure (for women having an ECV procedure) was measured immediately following the ECV using a single visual analogue scale with 0 representing ‘no pain at all’ and 100 representing ‘most pain imaginable’. Maternal satisfaction was determined using a structured questionnaire asking women if they would use the same approach to the timing of ECV in another pregnancy with a breech baby or to recommend it to a friend.

### Statistical analysis

#### Sample size

We estimated the rate of caesarean section in the delayed ECV group to be 65% based on rates from the pilot trial and adjusted for the change in the inclusion criteria. A sample size of 610/group was calculated to have 80% power to find an 8-percentage-point reduction in the rate of CS, from 65% in the delayed group to 57% in the early group (Type I error rate of 0.05; two-tailed), if such a reduction existed. This sample size would provide >70% power to detect an increase from 6% to 10% in the rate of preterm birth (Type I error rate of 0.05; two-tailed) and >85% power to detect a three-fold increase in the rate of perinatal or neonatal mortality or serious neonatal morbidity from 1.6% to 4.8%.

We then increased the sample size by 20%, initially to 1460 and finally to 1526, as we anticipated that approximately 20% of women in the early group would not have an ECV procedure because they would experience a spontaneous cephalic version after randomisation and before the scheduled version.

#### Interim analyses

Two planned safety interim analyses were conducted after complete data were received on the first 500 and 900 women randomised. Results were reviewed by an independent Data Safety and Monitoring Board blinded to group assignment.

#### Final analysis

An intention-to-treat analysis was conducted. Baseline data were compared descriptively between treatment groups. Perinatal and neonatal deaths were excluded from the analyses of measures of neonatal morbidity. Fisher's exact test was used to compare binary outcomes and Student's *t*-test to compare continuous variables that were normally distributed. Because only women with an ECV procedure were assessed for pain during the procedure, we used linear regression to calculate adjusted between-treatment comparisons of pain by controlling for baseline variables where there was either imbalance between treatment groups or where the variable correlated with pain scores on the first ECV. A *P*-value of <0.05 (two-sided) indicated statistical significance for the primary and secondary outcome and <0.01 (two-sided) indicated statistical significance for other outcomes. Relative risks and 95% CI were used to report the effects of the intervention on each outcome and risk difference (RD) was calculated for the primary and secondary outcomes. Subgroup analyses were undertaken using logistic regression analyses to test for interactions between baseline characteristics (parity [0 versus ≥1], type of breech [frank versus non-frank], gestational age at randomisation [33–34 weeks versus 35 weeks of gestation], and the national perinatal mortality rate of the country [≤20/1000 versus >20/1000]) and treatment group for the primary and secondary outcomes. The statistical software sas version 9.1 (SAS Institute, Cary, NC, USA) was used for the analyses.

In keeping with recommendations for presentation of findings in the context of prior research and existing knowledge,^14^ we undertook meta-analyses of the primary and secondary outcome data from this trial and the Early ECV Pilot Trial, the only previous study to compare early and delayed timing of ECV. We pooled the summary data for the primary and secondary outcomes of caesarean section and preterm birth before 37 weeks of gestation using a Mantel–Haenszel fixed effects model to calculate the RR, weighted RD and 95% CIs of harm or untoward outcome, using Review Manager (RevMan Version 5.0 Copenhagen: The Nordic Cochrane Centre, The Cochrane Collaboration, 2008) Using data from both trials, we calculated the number needed to treat to prevent one caesarean section and the number needed to treat to harm for preterm birth.

Steering committee. EK Hutton, J Barrett, GD Carson, MF Delisle, M Dunn, S Edwards, A Fernandez, A Gafni, ME Hannah, S Hewson, R Natale, A Ohlsson, SJ Ross, AR Willan, R Windrim, JK Pollard, I Schweitzer, G Sylvestre, P Turtle.

*Data Safety Monitoring Board.* Bracken M, Crowley P, Donner A, Duley L, Ehrenkranz R.

*Collaborators. Argentina*—M Curioni, R Abalos Gorostiaga (Hospital Ramon Carrillo, Santiago del Estero); C Becker, PA Elizabeth, L Errandonea, M Palermo, CA Ramos, M Trabucco, D Montes Varela (Hospital Posadas, Buenos Aires); MS Bertin, JL Castaldi (Hospital Penna, Bahia Blanca); M Mohedano de Duhalde (Hospital Avellaneda, Tucuman); C Becker (Hospital Durand, Buenos Aires); A Messina (Hospital Alvarez, Buenos Aires); *Australia*—J Baumgartner, G Kovacs, B Malcolm, JR Neil (Box Hill Hospital, Box Hill); K Mahomed (Ipswich Hospital, Ipswich); A Child, B DeVries, H Phipps, A Welsh (Royal Prince Alfred Hospital, Sydney); GK Davis, L Roberts, NP Watts (St George Hospital, Sydney); M Cybulski, D Gibson, S Tucker (Toowoomba Base Hospital, Toowoomba); I McCahon, P Sheehan, M Umstad (University of Melbourne Dept. of Obsetrics & Gynecology, The Royal Women's Hospital, Carlton); J Milligan, J Morris, K Rickard (Royal North Shore Hospital, St. Leonards); G Gardener, S Jenkins-Manning (Mater Mothers’ Hospital, Brisbane); C Boniface, M Edmondson, D Watson (Townsville Hospital, Townsville); A Green (Ipswich Hospital, Ipswich); *Brazil*—A Ayub (ISCMPA-Maternidade Mario Totta, Porto Alegre); *Canada*—MF Delisle, S Soanes, (Children's & Women's Health Centre of BC, Vancouver); A Jordan, R Windrim (Mount Sinai Hospital, Toronto); C Fanning, B Parish (IWK Health Centre, Halifax); R Natale, MA Watson (St Joseph's Health Centre, London); D Reid, P Scheufler (Trillium Health Centre, Mississauga); AM Malott, A Reitsma (Hamilton Health Sciences Corporation McMaster Site, Hamilton); KA Haslauer, M Lipp (Lion's Gate Hospital, Vancouver); D Farquharson, K Gray (Royal Columbian Hospital, New Westminster); N Demianczuk, E Penttinen (Royal Alexandra Hospital, Edmonton); E Herer, K McLean (Sunnybrook Health Sciences Centre, Toronto); F Aghajafari, S Williams (Regina General Hospital, Regina); C Moravac, M Yudin (St Michael's Hospital, Toronto); J Pollard, L Miller (Calgary Health Region—Foothills Hospital, Calgary); RB Anderson (St Paul's Hospital, Vancouver); M Good, MC Walker (The Ottawa Hospital—General Campus, Ottawa); R Kulkarni, R Scarfone (North Bay General Hospital, North Bay); C Cameron, T Peel (Markham Stouffville Hospital, Markham); *Chile*—J Carrillo, A Cruces, Y Gonzalez (Hospital Padre Hurtado, Santiago); J Figueroa Poblete, L Lama Hormazabal, J Saez, (Hospital Clinico San Borja Arriaran, Santiago); E Oyarzun, A Rioseco (Pontificia Universidad Católica de Chile, Santiago); S Illanes, C Kottmann (Hospital Parroquial de San Bernardo, San Bernardo); M Parra, S Quezada, L Quiroz (Hospital Clinico Universidad de Chile JJ Aguirre, Santiago); *Denmark*—L Hvidman, IM Mogensen, A Mouritzen (Aarhus University Hospital, Aarhus); B Ostberg (Gentofte University Hospital, Hellerup); *Egypt*—SNM Abdel-Samad, T Al-Hussaini, I El-Nashar (Assiut University Hospital, Assiut); *Estonia*—F Kirss, K Rull, E Ustav, P Vaas (Tartu University Hospital-Women's Clinic, Tartu); *Germany*—V Brink-Spalink, K Weizsaecker (CUB-Virchow Klinikum, Berlin); *Hungary*—T Major, R Poka (University of Debrecen, Debrecen); *Ireland*—S Daly, (Coombe Women's Hospital, Dublin); *Israel*—H Kaneti, D Rosen, B Schachter (Meir Medical Center, Kfar-Saba); B Chayen, L Harel (Ma'ayney HaYeshua Medical Center, Bnei Brak); Z Hiaeb, G Malinger (Edith Wolfson Medical Center, Holon); D Dukler, E Lunenfeld (Soroka Medical Center, Beer Sheva); *Jordan*—L AlFaris, M El-Zibdeh (Islamic Hospital, Amman); *Poland*—I Domzalska-Popadiuk, P Kobiela, Z Pankrac, J Preis, K Preis, M Swiatkowska-Freund (Medical University of Gdansk, Gdansk); Portugal—J Cravo (Hospital Distrital de Faro, Faro); *South Africa*—AM Theron, GB Theron (Stellenbosch University, Tygerberg); HS Cronje, JM du Plessis (University of Free State, Bloemfontein); *Spain*—M Munoz (Hospital Clinic-University of Barcelona, Barcelona); *Sultanate of Oman*—G Khan, S Khan (Khoula Hospital, Muscat); *the Netherlands*—S Goossens, M Pieters, FJME Roumen (Atrium Medical Center, Heerlen); F ten Cate, M Pieters, F Smits (Academisch Ziekenhuis Maastricht, Maastricht); M Heres, E Krabbendam (Sint Lucas Andreas Ziekenhuis, Amsterdam); *United Kingdom*—R Airey, D Farrar, DJ Tuffnell (Bradford Royal Infirmary, Bradford); V Heyes, C Melvin, C Schram (Royal Blackburn Hospital, Blackburn); A Galimberti, P Stewart (Royal Hallamshire Hospital, Sheffield); J Cresswell (Chesterfield Royal Hospital, Chesterfield); C McCormick (Nottingham City Hospital, Nottingham); *United States of America*—J Andrews, D Fleener (University of Iowa Hospitals and Clinics, Iowa City); D Coonrod, BF Jimenez (Maricopa Medical Center, Phoenix); S Brown, A Gregg (University of South Carolina, Columbia); C Pitchford, D Seubert (New York University School of Medicine, New York).

## Results

A total of 1543 women were randomised from 68 centres in 21 countries between 30 December 2004 and 25 June 2008 (see Appendix S1). Two women, one in each group, asked to be removed from the study leaving 1541 women for the analyses of baseline characteristics; 767 in the early ECV group, and 774 in the delayed ECV group (Figure 1). Eight women were lost to follow up (two assigned to early ECV and six assigned to delayed ECV); seven before any intervention, and one following a first ECV procedure. This left 1533 women (99.4%) for the analysis of maternal outcomes; 765 in the early ECV group, 768 in the delayed ECV group.

Baseline characteristics were similar in the two groups (Table 2). The timing and number of ECV procedures including success of the procedure and rates of spontaneous version are presented in Table 3. Of the 765 women randomised to the early ECV group, 615 (80.4%) underwent at least one ECV procedure versus 461 (60.0%) of 769 women in the delayed ECV group. The majority of women in both groups underwent only one procedure. Of 615 women having an ECV procedure in the early ECV group, 584 (95.0%) had their first procedure at the expected time between 34^0/7^ and 35^6/7^ weeks of gestation; of 461 women having an ECV procedure in the delayed ECV group, 430 (93.3%) had their first ECV procedure at the expected time at or after 37^0/7^ weeks of gestation.

**Table 2:** Baseline characteristics

Characteristic at randomisation	Early ECV (*n* = 767)*n* (%)	Delayed ECV (*n* = 774)*n* (%)
**Maternal age** (years) (median 5th, 95th centile)	30.0 (18.9, 39.2)	30.0 (19.8, 39.2)
**Parity**		
0	409 (53.3)	411 (53.1)
1–4	346 (45.1)	354 (45.7)
>4	12 (1.6)	9 (1.2)
**Gestational age** (weeks) Median (5th, 95th centile)	34.7 (33.1, 35.7)	34.9 (33.3, 35.7)
33^0^–33^6^	131 (17.1)	109 (14.1)
34^0^–34^6^	318 (41.5)	323 (41.7)
35^0^–35^6^	318 (41.5)	342 (44.2)
Method of determining gestational age*		
Clinical history only (no ultrasound)	61 (8.0)	49 (6.4)
First ultrasound ≤20 weeks (± clinical history)	627 (81.9)	656 (85.2)
First ultrasound >20 weeks (± clinical history)	78 (10.2)	65 (8.4)
**Maternal height** (cm) (median 5th, 95th centile)†	163.0 (151.0, 176.0)	163.0 (152.0, 176.0)
**Maternal weight at last prenatal visit** (kg)§ Median (5, 95th centile)	74.0 (56.0, 104.5)	72.0 (54.4, 100.0)
**One previous caesarean section**¶	36 (4.7)	29 (3.8)
**Time from last ultrasound to randomisation ≤7** (**days**)**	757 (99.0)	768 (99.6)
**Placental location anterior**‡	314 (41.0)	319 (41.4)
**Type of breech at last ultrasound**§§		
Frank	475 (62.1)	467 (61.0)
Complete	247 (32.3)	253 (33.0)
Footling	43 (5.6)	46 (6.0)
**National perinatal mortality rate**¶¶		
≤10/1000	656 (85.5)	664 (85.8)
>10–20/1000	61 (8.0)	62 (8.0)
>20/1000	50 (6.5)	48 (6.2)

Missing values in early, delayed group as follows: *1,4; †1,4; §13,9; ¶1,1; **1,3; ‡1,3; §§2, 8.

¶¶Countries with a national perinatal mortality rate of ≤10/1000 were Australia, Canada, Chile, Denmark, Estonia, Germany, Hungary, Ireland, Israel, The Netherlands, Poland, Portugal, Spain, UK, USA; countries with a national perinatal mortality rate of >10–20/1000 were Argentina, Brazil, Oman; countries with a national perinatal mortality rate of >20/1000 were Egypt, Jordan, South Africa; Reference for National Perinatal Mortality Rates: Neonatal and perinatal mortality: country, regional and global estimates. World Health Organization. Geneva 2006.

**Table 3:** Description of ECV procedures

Description of procedures	Early ECV*n* (%)	Delayed ECV*n* (%)
	*n* = 765	*n* = 769

**Time from randomisation to first ECV procedure**		
≤7 days	571 (74.6)	10 (1.3)
>7 days	44 (5.8)	451 (58.7)
No ECV procedure undertaken	150 (19.6)	308 (40.1)
Reasons for no ECV procedure*		
Spontaneous version	106 (13.9)	194 (25.2)
Mother declined ECV	29 (3.8)	47 (6.1)
Developed contraindication to ECV	14 (1.8)	33 (4.3)
Mother delivered before ECV	1 (0.1)	34 (4.4)
Clinician declined	3 (0.4)	6 (0.8)
Logistics	2 (0.3)	1 (0.1)
Other†	0	2 (0.3)
**ECV procedure**	615 (80.4)	461 (60.0)

	*n* = 615	*n* = 461

Gestational age at first ECV procedure		
Median (weeks) (5–95th centile)	35.1 (34.0, 35.9)	37.3 (36.9, 37.9)
<34^0/7^	14 (2.3)	0 (0.0)
34^0/7^–35^6/7^	584 (95.0)	5 (1.1)
36^0/7^–36^6/7^	12 (2.0)	26 (5.6)
≥37^0/7^	5 (0.8)	430 (93.3)
Fetal presentation before first ECV procedure		
Frank breech	372 (60.5)	275 (59.7)
Complete breech	189 (30.7)	144 (31.2)
Footling breech	31 (5.0)	24 (5.2)
Unknown breech	6 (1.0)	1 (0.2)
Transverse/oblique lie	17 (2.8)	17 (3.7)
Position of breech at first ECV procedure		
Sacrum anterior	120 (20.1)	91 (20.5)
Sacrum posterior	71 (11.9)	47 (10.6)
Sacrum transverse	353 (59.0)	266 (60.0)
Unknown	54 (9.0)	40 (9.0)
Station of presenting part before first ECV procedure		
Floating	236 (38.4)	121 (26.3)
Dipping	244 (39.7)	209 (45.3)
Well into the pelvis orengaged	122 (19.8)	124 (26.9)
Unknown	13 (2.1)	7 (1.5)
Fetus easily palpated before first ECV procedure		
Yes	518 (84.2)	414 (89.8)
No	94 (15.3)	45 (9.8)
Unknown	3 (0.5)	2 (0.4)
Use of tocolytics for ECV		
During all ECV procedures	419 (68.1)	316 (68.6)
During some ECV procedures	10 (1.6)	8 (1.7)
During no ECV procedures	186 (30.2)	136 (29.5)
Tocolytics used*		
Betamimetic	335 (54.5)	252 (54.7)
Nitric oxide donor	36 (5.9)	23 (5.0)
Oxytocin antagonist	29 (4.7)	30 (6.5)
Calcium channel blocker	27 (4.4)	15 (3.3)
Prostaglandin synthetase inhibitor	19 (3.1)	20 (4.3)
Magnesium sulphate	0	1 (0.2)
Use of epidural for one or more procedures	0 (0.0)	0 (0.0)
Use of spinal for one or more procedures	1 (0.2)	0 (0.0)
Number having same spinal for delivery	0 (0.0)	0 (0.0)
**Success of ECV procedures**		
Any ECV procedure successful	329 (53.5)	201 (43.6)
Only one procedure undertaken	536 (87.2)	426 (92.4)
First procedure successful	316 (51.4)	194 (42.1)
Two procedures undertaken	73 (11.3)	34 (7.4)
Second procedure successful	22	7
More than two procedures undertaken	6 (1.0)	0 (0.0)
Third or subsequent procedure successful	3	
Ease with which first successful ECV performed§		
Very easy	104 (32.9)	43 (22.2)
Somewhat easy	121 (38.3)	81 (41.8)
Neither easy nor difficult	55 (17.4)	40 (20.6)
Somewhat difficult	29 (9.2)	27 (13.9)
Very difficult	6 (1.9)	3 (1.6)
Reasons for discontinuing first ECV*		
Unable to turn fetus	211 (34.3)	187 (40.6)
Unable to lift breech frompelvis	97 (15.8)	93 (20.2)
Maternal discomfort	74 (12.0)	61 (13.2)
Non-reassuring fetal heartrate	11 (1.8)	11 (2.4)
Obese/unable to palpate	11 (1.8)	5 (1.1)
Other	4 (0.7)	6 (1.3)
Maternal and fetal complications during ECV*		
Non reassuring fetal heartrate	21 (3.4)	16 (3.5)
Contractions	1 (0.2)	2 (0.4)
Hypotension	1 (0.2)	2 (0.4)
Vaginal bleeding/suspected abruption placenta	0	1 (0.2)
Fetal presentation after first ECV		
Cephalic	316 (51.4)	194 (42.1)
Breech	283 (46.0)	250 (54.2)
Transverse lie	16 (2.6)	17 (3.7)
Presentation of fetus after final ECV		
Cephalic	328 (53.3)	200 (43.4)
Breech	275 (44.7)	246 (53.4)
Transverse lie	12 (2.0)	14 (3.0)
**Pain during first ECV**¶		
Adjusted Mean (SE)**	40.6 (1.06)	45.2 (1.2)

* May be more than one reason given.

† Other reasons in delayed group included an unstable lie and an unfavourable position for ECV with spines posterior.

§ One missing value for early group.

¶ Fifteen missing values in the early group; six missing values in the delayed group.

** Adjusted for parity, maternal age and weight, and anterior placenta; *P* = 0.0001.

All ECV procedures were performed in hospital with almost all on or near the labour and birth delivery area (563/615 [91.5%] in the early ECV group and 414/460 [90.0%] in the delayed ECV group) and by an experienced practitioner as determined *a priori* (603/615 [98.1%] in the early ECV group and 446/461 [96.8%] in the delayed ECV group). Clinician experience in both groups was similar and the majority of ECV procedures were undertaken by clinicians with >10 years experience with ECV: early ECV group 343/615 (55.8%) and delayed ECV group 255/461 (55.3%). Obstetricians performed most ECV procedures with midwives performing the remaining procedures; 2.1% (13/615) and 2.6% (12/461) in the early and delayed groups, respectively.

The characteristics of the fetuses at the time of the first ECV procedure for those women having an ECV procedure are reported in Table 3, as are the outcomes of the ECV procedures. The rate of any maternal and fetal complications encountered during any ECV procedure was low: (21/615 [3.4%] in the early ECV group and 20/461 [4.3%] in the delayed ECV group), with non-reassuring fetal heart rate during the procedure being the complication that occurred most frequently. Women in the early ECV group experienced less pain during the first ECV procedure than those in the delayed ECV group, after adjusting for parity, maternal age and weight, and anterior placenta (adjusted mean score 40.6 [standard error (SE) 1.06] compared with 45.2 [SE 1.2], *P* = 0.0001).

Most infants in both groups were born in hospital and women had a median postpartum hospital stay of 68 hours (Table 4).

**Table 4:** Characteristics and outcomes of pregnancy, labour and birth

Characteristic or outcome	Early ECV (*n* = 765)*n* (%)	Delayed ECV (*n* = 768)*n* (%)
Presentation at delivery*		
Cephalic	451 (59.0)	391 (50.9)
Non-cephalic	314 (41.1)	377 (49.1)
**Caesarean section**†	398 (52.0)	430 (56.0)
Parity = 0	286/409 (69.9)	294/406 (72.4)
Parity = 0 and cephalic at birth	52/174 (29.9)	40/148 (27.0)
Parity ≥1	112/356 (31.4)	136/361 (37.7)
Parity ≥1 and cephalic at birth	36/277 (13.0)	25/242 (10.3)
**Caesarean section**†	398 (52.0)	430 (56.0)
Before labour or in early labour	285 (37.3)	338 (44.0)
During active first§ or second stage labour	113 (14.8)	92 (12.0)
**Vaginal birth**	367 (48.0)	338 (44.0)
Spontaneous cephalic	318 (41.6)	287 (37.4)
Assisted cephalic (vacuum or forceps)	45 (5.9)	39 (5.1)
Vaginal breech¶	4 (0.5)	12 (1.6)
**Place of birth**		
Hospital	751 (98.2)	734 (95.6)
Home, birthing centre, or other**	14 (1.8)	34 (4.4)
**Gestational age at delivery** (median, 5th, 95th centile)	39.1 (36.6, 41.6)	39.1 (37.1, 41.4)
**Preterm birth <37 weeks**‡	50 (6.5)	34 (4.4)
Gestational age <34 weeks	0	1 (0.1)
Gestational age 34^0^–34^6^ weeks	5 (0.7)	1 (0.1)
Gestational age 35^0^–35^6^ weeks	17 (2.2)	13 (1.7)
Gestational age 36^0^–36^6^ weeks	28 (3.7)	19 (2.5)
**Birth within 48 hours of ECV procedure**	7 (1.1)	1 (0.2)
**Serious fetal complications following randomisation**		
Prelabour rupture of membranes <37 weeks	12 (1.6)	9 (1.2)
Abruptio placenta requiring intervention to effect delivery	3 (0.4)	2 (0.3)
Preterm labour resulting in preterm birth	26 (3.4)	17 (2.2)
FHR abnormalities before labour requiring delivery	10 (1.3)	9 (1.2)
**At least one serious fetal complication following randomisation**§§	41 (5.4)	31 (4.0)
**At least one serious fetal complications within 48 hours of ECV**	9/615 (1.5)	3/461 (0.7)
**Other fetal outcomes**		
Prelabour rupture of membranes	201 (26.3)	180 (23.4)
Placental abruption	10 (1.3)	5 (0.7)
Preterm labour	35 (4.6)	26 (3.4)
FHR abnormalities before labour (*n*)	28 (3.7)	23 (3.0)
**Maternal mortality or any serious maternal morbidity**¶¶during pregnancy, labour, birth or up to 28 days following birth***	39 (5.1)	29 (3.8)
**Duration of postpartum stay in hours** (median, 5th, 95th centile)	67.9 (9.2, 123.5)	67.8 (5.8, 124.4)

ECV = External Cephalic Version;

* RR = 0.84; 95% CI: 0.75, 0.94; p-value = 0.002;

† RR = 0.93; 95% CI: 0.85, 1.02; p-value = 0.12;

§ Active labour is defined as cervical dilation ≥3 cm or 80% effaced and contractions ≤5 minutes apart; no significant interaction between treatment group and the baseline variables parity, type of breech, gestational age at randomisation and national perinatal mortality rate;

¶ All vaginal breeches reported as assisted, no forceps for aftercoming head and no breech extraction;

** Other two locations in delayed group include ambulance and unplanned out-of-hopsital birth;

‡ RR = 1.48; 95% CI: 0.97, 2.26; p-value = 0.07; no significant interaction between treatment group and the baseline variables parity, type of breech, gestational age at randomisation and national perinatal mortality rate;

§§ RR = 1.33; 95% CI: 0.84, 2.09; p-value = 0.23;

¶¶ There were no maternal deaths or any cases of fistula involving the genital tract, sympotomatic broad ligament haematoma, pulmonary embolism, adult respiratory distress syndrome, or amniotic fluid embolism.

*** RR = 1.35; 95% CI 0.84, 2.16; p-value = 0.22.

Fewer infants were in a non-cephalic presentation at birth in the early ECV group than the delayed ECV group (314/765 [41.1%] versus 377/768 [49.1%]; RR 0.84, 95% CI 0.75, 0.94; *P* = 0.002). The rate of caesarean section was not different between groups (398/765 [52.0%] in the early ECV group versus 430/768 [56.0%] in the delayed ECV group; RR 0.93, 95% CI 0.85, 1.02; *P* = 0.12; RD −0.04, 95% CI −0.09, 0.01). The rate of preterm birth at <37 weeks of gestation was not different between groups (6.5% [50/765] in the early ECV group, 4.4% [34/768] in the delayed ECV group, RR 1.48, 95% CI 0.97, 2.26; *P* = 0.07; RD −0.02, 95% CI −0.00, 0.04). There was no difference between groups in the rate of one or more serious fetal complications following randomisation (41/765 [5.4%] in the early ECV group versus 31/768 [4.0%] in the delayed ECV group; RR 1.33, 95% CI 0.84, 2.09; *P* = 0.23). There were no maternal deaths in either group and no difference between groups in serious maternal morbidity (39/765 [5.1%] in the early ECV group versus 29/768 [3.8%] in the delayed ECV group; RR 1.35, 95% CI 0.84, 2.16; *P* = 0.22) (Table 4 and Appendix S2).

One infant in the early ECV group was excluded from the analysis of perinatal/neonatal outcomes because of a lethal anomaly (trisomy 18), leaving 764 infants in the early ECV group and 768 infants in the delayed ECV group for the analyses of perinatal and neonatal outcomes (Table 5). Three infants died, all in the delayed ECV group, none of whom had had an ECV procedure. The causes of death were unexplained intrauterine fetal death at a gestational age of 37 weeks 5 days; sepsis following cardiac surgery; and sepsis after preterm birth. One infant in each group had serious neonatal morbidity. Perinatal or neonatal mortality or serious neonatal morbidity was not different between the groups (1/764 [0.1%] in the early ECV group versus 4/768 [0.5%] in the delayed ECV group, RR 0.25, 95% CI 0.03, 2.25; *P* = 0.37). There were no reported cases of intubation and ventilation via endotracheal tube for 24 hours or longer, bronchopulmonary dysplasia, seizures within 72 hours of age, necrotising enterocolitis, birth trauma, grade III or IV intraventricular haemorrhage, cystic periventricular leucomalacia, or cases with evidence of stroke. The risk of being admitted to neonatal intensive care for ≥24 hours was not different between groups (5/764 [0.7%] in the early ECV group versus 6/765 [0.8%] in the delayed ECV group, RR 0.83, 95% CI 0.26, 2.72; *P* = 1.00).

**Table 5:** Neonatal outcomes

Outcome	Early ECV *n* (%)	Delayed ECV *n* (%)
	(*n* = 764)	(*n* = 768)

Perinatal or neonatal mortality or serious neonatal morbidity*,†	1 (0.1)	4 (0.5)
**Perinatal or neonatal death 0–27 days after birth**§	0 (0.0)	3 (0.4)
Stillbirth	0 (0.0)	1 (0.1)
Neonatal death	0 (0.0)	2 (0.3)

	**(*n* = 764)**	**(*n* = 765)**
**Serious neonatal morbidity**¶	1 (0.1)	1 (0.1)
Apgar score <4 at 5 minutes**	0 (0.0)	1 (0.1)
Sepsis within 72 hours of birth – confirmed by blood culture	1 (0.1)	0 (0.0)
**Other neonatal morbidity**¶		
Apgar score <7 at 5 minutes**	6 (0.8)	4 (0.5)
Intubation and ventilation	5 (0.7)	2 (0.3)
**Admission to neonatal intensive care for ≥24 hours‡**	5 (0.7)	6 (0.8)
**Other neonatal outcomes**		
Birthweight		
Median (5th, 95th centile)(grams)	3340 (2475, 4120)	3330 (2590, 4110)
<2500 g	39 (5.1)	24 (3.1)
>4000 g	67 (8.8)	61 (8.0)
Gender		
Male	362 (47.4)	366 (47.8)
Female	402 (52.6)	399 (52.2)

* RR = 0.25; 95% CI 0.03, 2.25; *P* = 0.37

† There were no reported cases of: birth trauma; abnormal level of consciousness that included coma, stupor or decreased response to pain; neonatal seizures before 72 hours of age; need for assisted ventilation ≥24 hours via endotracheal tube initiated within 72 hours after birth; meningitis within 72 hours after birth; necrotising enterocolitis; bronchopulmonary dysplasia; Grade III or IV intraventricular haemorrhage; or cystic periventricular leucomalacia

§ One term infant died at 17 days from sepsis following cardiac surgery; one 2570-g infant born at 35 weeks 1 day died on day 11 of *Klebsiella pneumoniae* after being discharged home and readmitted to NICU; one was an unexplained stillbirth born at 37 weeks 5 days; none of the three women had an ECV procedure.

¶ Excludes deaths.

** Missing data for two women in the early group, one woman in the delayed group; for these women, there was no noted resuscitation at time of delivery and no other complications noted.

‡ RR = 0.83, 95% CI 0.26, 2.72; *P* = 1.00.

Women in the early ECV group were more likely than those in the delayed ECV group to indicate that they would use the same approach to the timing of ECV in another pregnancy with a fetus in breech presentation or to recommend it to a friend (630/723 [87.1%] versus 531/710 [74.8%], RR 1.17; 95% CI 1.11, 1.23, *P* < 0.0001).

For outcomes of caesarean section and preterm birth, there were no significant interactions between treatment group and the baseline variables parity, type of breech, gestational age at randomisation and national perinatal mortality rate. As a result, there were no subgroup effects observed for these outcomes.

**Figure 2:**
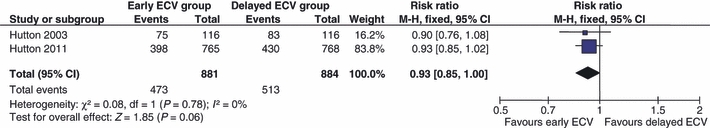
Forest plot comparison: meta-analysis of caesarean section.

**Figure 3:**
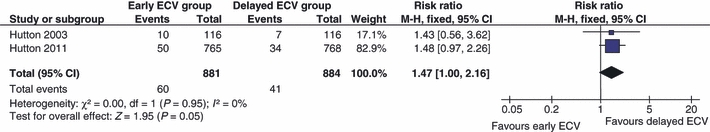
Forest plot comparison: meta-analysis of preterm birth <37 weeks of gestation.

When the data from this trial were combined with those of the pilot trial we found no evidence of heterogeneity (*I*^2^ = 0%). The caesarean section rate in the early ECV group was 53.7% (473/881) compared with 58.1% (513/884) in the delayed ECV group (RR 0.93, 95% CI 0.85, 1.00, *P* = 0.06; RD −0.04, 95% CI −0.09, 0.00, *P* = 0.06) (Figure 2). The rate of preterm birth in the early ECV group was 6.8% (60/881) compared with 4.6% (41/884) in the delayed group (RR 1.47, 95% CI 1.00, 2.16, *P* = 0.05; RD 0.02, 95% CI −0.00, 0.04) (Figure 3). The differences between groups in the rates of both caesarean section and preterm birth are at borderline conventional levels of statistical significance. We calculated that 23 women (95% CI 11 to undefined) would need to plan early ECV procedures to prevent one caesarean section, and for every 46 (95% CI 23 to undefined) planned early ECV procedures, one additional late preterm birth would result. In both these calculations, because the risk difference is not statistically significant at the 5% level (two-sided), the upper limit is undefined and can be considered arbitrarily large.

## Discussion

When the fetus presents as a breech, ECV undertaken at term reduces the need for caesarean section, avoids the risk of preterm birth and is considered safe for the fetus.^3^,^6^,^7^,^15^,^16^ However, because of the risk of a failed procedure when ECV is not undertaken until 37 weeks of gestation, we hypothesised that initiating ECV somewhat earlier than term might increase the likelihood of successful ECV, thereby further reducing the need for caesarean section.^17^–^19^

The Early ECV2 Trial found that initiating ECV at 34^0/7^–35^6/7^ weeks of gestation, rather than waiting until term, was successful at decreasing the likelihood of a non-cephalic presentation at birth. However, the decrease in non-cephalic presentation did not translate into a reduction in use of caesarean section. The results of the meta-analysis, although at borderline conventional levels of statistical significance, suggest that the small (4%) reduction in absolute risk of caesarean section that we found in this trial with early versus delayed ECV may be real. For women with a fetus in breech presentation who wish to reduce their risk of caesarean section, even if it is only by this small percentage, ECV at 34–35 weeks of gestation is an option that may be considered.

Although our trial did not find higher risks of adverse outcome for the fetus with early versus delayed ECV and generally the infants did well, the results of the meta-analysis suggest that early ECV may be associated with a higher risk of preterm birth. This may be because the manipulation of the uterus in attempting to turn the fetus as part of ECV contributes to initiating labour or results in a fetal complication which, in the early group, may lead to preterm birth. Irrespective of the cause, preterm birth, even at 34–36 weeks of gestation, is a concern because it is associated with acute morbidities such as respiratory distress syndrome as well as poorer long-term outcomes.^20^,^21^ Hence any benefits to undertaking the ECV before term in avoiding caesarean section will need to be balanced against the potentially higher risk of preterm birth.

Pain scores for the ECV procedure in both groups were moderate. Although the early group perceived less pain, it is unlikely that a 5mm difference on the 100mm visual analogue scale is clinically important. For example, in an emergency room setting a difference of <13 mm in pain scores on the visual analogue scale was not found to be clinically important.^22^ Women were more satisfied with early ECV than delayed ECV in this trial. This may be because of the perceived benefits of a lower risk of caesarean section or because the early procedure was not generally available outside the trial and so some of the women joining the trial may have been doing this to have a chance of receiving the treatment that they preferred. Further research would be useful to explore women's views about the timing of ECV, given the findings from this trial.

It is not clear why the decrease in non-cephalic presentation did not result in decreased rates of caesarean section. It may be because more breech fetuses in the delayed group were born vaginally; the study was undertaken in centres where the rates of caesarean section for cephalic presentation are generally high; and because the slightly higher rate of preterm fetal complications in the Early ECV Group may have contributed to a greater need for caesarean section in that group despite the fetus being in cephalic presentation.

Tocolytics are often used as part of the ECV procedure and are known to increase the success of the procedure.^23^ As such tocolytics are the most important co-intervention to consider in this trial. We believe that our study reflects the use of tocolytics in practice; that is some practitioners use tocolytics routinely, some use them selectively and some do not use tocolytics at all for ECV. It is, for example, the case in Canada that betamimetics are not available for ECV. In our trial, 70% of the ECV procedures in both groups were carried out using tocolytics; so the use of tocolytics was balanced between groups averting any co-intervention bias.

Lack of power limits the ability to conclude that a real difference in caesarean section rates between groups exists. The original total sample size of 1460 was based on having a power of 80% to detect an 8-percentage-point difference, assuming the caesarean section rate in the delayed group was 65%. For a caesarean section rate of 56%, as observed in the trial, a total sample size of 1460 provides a power of 78%. However, as 1524 women were enrolled, a power of 80% was maintained. Although we calculated our sample size using data from our pilot study, The Early ECV2 Trial had a smaller than anticipated between-group difference in rate of caesarean section (8% in sample size calculations versus 4% in trial) which undermined the power to find a difference in rate of caesarean section. The rate of caesarean section may have been lower in the current study because the proportion of multiparous women enrolled was higher (47%) than in the pilot study (35%), and multiparity is associated with higher rates of spontaneous version of breech to cephalic, of successful ECV and of vaginal birth. We also had a 1% vaginal breech rate that was not accounted for in the sample size calculation. In addition, vaginal breech births were not evenly distributed between the groups with the early ECV group having 4/765 (0.5%) compared with 12/768 (1.6%) in the delayed ECV group. These factors all have the potential to decrease statistical power. Compliance with the study protocol was generally good, however 6.7% (31/461) of the ECV procedures in the delayed group were undertaken before 37 weeks of gestation, usually as part of a clinical management plan for a condition that arose following randomisation.

Factors that affect external validity and generalisability of findings in randomised controlled trials have been well enumerated.^24^,^25^ The number of women enrolled in the Early ECV2 Trial was low relative to potentially eligible women. This may be partly explained by the fact that ECV is not offered or recommended to women as frequently as it should be according to current recommendations,^3^–^5^ and when it is offered, women are often fearful of the pain and safety of the procedure.^26^ Potentially eligible women may not have been identified in time to enrol in our study, as not all clinicians focus on fetal presentation as early as 34–36 weeks of gestation. In addition, recruitment is often difficult, generally, to randomised controlled trials. Despite the proportionately low numbers of women recruited, we believe that our sample was probably representative, as the rate of successful ECV in our study groups (54% and 44% for early and delayed groups, respectively) was similar to the mean rate found in two large systematic reviews reporting on ECV outcomes (60% and 58%).^6^,^7^ Our multicentred pragmatic trial design will enhance the generalisability of the findings. The entry criteria were broad and inclusive and practitioners undertook the ECV according to their local protocol.

## Conclusion

External cephalic version initiated at 34–35 weeks of gestation compared with 37 or more weeks of gestation increases the probability of cephalic presentation at birth, but does not reduce the rate of caesarean section and may increase the rate of preterm birth. The study findings should be discussed with women so that they can make an informed choice as to what is best for them and their infants.

## EECV2 Collaborative Group

### Disclosure of interest

No author had any financial or personal relationships with other people or organisations that could inappropriately influence (bias) their work on this project. The funding sponsor The Canadian Institutes of Health Research (CIHR) had no role in the collection, analysis or interpretation of data; in the writing of the report; or decision to submit findings for publication. A staff member of CIHR sat as *ex officio* member of the Steering Committee for the trial.

### Contribution to authorship

All authors participated in the design, methodological issues implementation, conduct, monitoring, analysis, interpretation of the study, and participated in reviewing and editing the manuscript. The corresponding author had full access to all the data in the study and had final responsibility for the decision to submit for publication.

### Details of ethics approval

Ethics approval was obtained from each of three co-ordinating centres (University of British Columbia; Sunnybrook and Women's Health Sciences Centre; McMaster University), and at each of the recruiting centres where the study took place. In addition, informed consent was sought from each woman enrolled in the trial.

### Funding

EECV2 was funded by The Canadian Institutes of Health Research (MTC-65630) and registered with Clinical Trials.gov (ISRCTN 56498577). The data co-ordinating centre was supported by grants from Sunnybrook Health Sciences Centre, Women's College Hospital and the Department of Obstetrics and Gynaecology at the University of Toronto. Eileen Hutton was supported by salary awards from The Canadian Institutes of Health Research and from Michael Smith Foundation for Health Research.

## Supporting information

The following supplementary materials are available for this article:

**Appendix S1.** EECV2 Trial: final trial recruitment, annual delivery rate and number of certified ECV practitioners by participating site.

**Appendix S2.** Maternal mortality and morbidity.
