# Supplementary material for: The Early External Cephalic Version (ECV) 2 Trial: an international multicentre randomised controlled trial of timing of ECV for breech pregnancies
Source: BJOG. 2011 Feb 4;118(5):564–77. doi: 10.1111/j.1471-0528.2010.02837.x (PMC3085121; doi:10.1111/j.1471-0528.2010.02837.x)
Supplement: Supplementary file 1 [file bjo0118-0564-SD1.doc]

**Supporting Information**

**Appendix S1. EECV2 Trial: final trial recruitment, annual delivery rate and number of certified ECV practitioners by participating site**

| **Centre name, city and country** | **Collaborators** | **Number recruited to EECV2 Trial*** | **Number of deliveries at site per year** | **Number experienced practitioners**** **determined a priori** |
| --- | --- | --- | --- | --- |
| ***Argentina*** |  |  |  |  |
| Hospital Ramon Carrillo, Santiago del Estero | M Curioni, R Abalos Gorostiaga | 28 | 6500 | 4 |
| Hospital Posadas, Buenos Aires | C Becker, PA Elizabeth, L Errandonea, M Palermo, CA Ramos, M Trabucco, D Montes Varela | 26 | 4100 | 8 |
| Hospital Penna, Bahia Blanca | MS Bertin, JL Castaldi | 13 | 2700 | 6 |
| Hospital Avellaneda, Tucuman | M Mohedano, SR Rojas | 11 | 4000 | 6 |
| Hospital Durand, Buenos Aires | C Becker | 7 | 1800 | 4 |
| Hospital Alvarez, Buenos Aires | A Messina | 6 | 1500 | 9 |
| ***Australia*** |  |  |  |  |
| Box Hill Hospital, Box Hill | J Baumgartner, G Kovacs, B Malcolm, JR Neil | 117 | 3550 | 3 |
| Ipswich Hospital, Ipswich | K Mahomed, A Green | 59 | 2000 | 10 |
| Royal Prince Alfred Hospital, Sydney | A Child, B DeVries, H Phipps, A Welsh | 52 | 4400 | 3 |
| St George Hospital, Sydney | GK Davis, L Roberts, NP Watts | 38 | 2200 | 3 |
| Toowoomba Base Hospital, Toowoomba | M Cybulski, D Gibson, S Tucker | 24 | 1600 | 5 |
| University of Melbourne Dept. of Obsetrics & Gynecology, The Royal Women's Hospital, Carlton | I McCahon, P Sheehan, M Umstad | 19 | 4500 | 1 |
| Royal North Shore Hospital, St. Leonards | J Milligan, J Morris, K Rickard | 13 | 5000 | 2 |
| Mater Mothers' Hospital, Brisbane | G Gardener, S Jenkins-Manning | 11 | 7900 | 1 |
| Townsville Hospital, Townsville | C Boniface, M Edmondson, D Watson | 5 | 1500 | 5 |
| ***Brazil*** |  |  |  |  |
| ISCMPA-Maternidade Mario Totta, Porto Alegre | A Ayub | 1 | 4300 | 7 |
| ***Canada*** |  |  |  |  |
| Children's & Women's Health Centre of BC, Vancouver | MF Delisle, S Soanes | 40 | 7500 | 5 |
| Mount Sinai Hospital, Toronto | A Jordan, R Windrim | 39 | 6500 | 8 |
| IWK Health Centre, Halifax | C Fanning, B Parish | 23 | 4600 | 12 |
| St Joseph's Health Centre, London | R Natale, MA Watson | 22 | 6000 | 4 |
| Trillium Health Centre, Mississauga | D Reid, P Scheufler | 19 | 4200 | 4 |
| Hamilton Health Sciences Corporation McMaster Site, Hamilton | AM Malott, A Reitsma | 12 | 3200 | 9 |
| Lion's Gate Hospital, Vancouver | KA Haslauer, M Lipp | 12 | 1400 | 7 |
| Royal Columbian Hospital, New Westminster | D Farquharson, K Gray | 12 | 2800 | 7 |
| Royal Alexandra Hospital, Edmonton | N Demianczuk, E Penttinen | 11 | 4000 | 5 |
| Sunnybrook Health Sciences Centre, Toronto | E Herer, K McLean | 10 | 3500 | 11 |
| Regina General Hospital, Regina | F Aghajafari, S Williams | 9 | 2950 | 4 |
| St Michael's Hospital, Toronto | C Moravac, M Yudin | 9 | 2600 | 9 |
| Calgary Health Region – Foothills Hospital, Calgary | J Pollard, L Miller | 7 | 10000 | 16 |
| St Paul’s Hospital, Vancouver | RB Anderson | 7 | 1700 | 7 |
| The Ottawa Hospital – General Campus, Ottawa | M Good, MC Walker | 7 | 3800 | 7 |
| North Bay General Hospital, North Bay | R Kulkarni, R Scarfone | 6 | 850 | 4 |
| Markham Stouffville Hospital, Markham | C Cameron, T Peel | 5 | 2820 | 8 |
| ***Chile*** |  |  |  |  |
| Hospital Padre Hurtado, Santiago | J Carrillo, A Cruces, Y Gonzalez | 106 | 6200 | 3 |
| Hospital Clinico San Borja Arriaran, Santiago | J Figueroa Poblete, L Lama Hormazabal, J Saez | 86 | 7500 | 3 |
| Pontificia Universidad Católica de Chile, Santiago | E Oyarzun, A Rioseco | 39 | 2200 | 10 |
| Hospital Parroquial de San Bernardo, San Bernardo | S Illanes, C Kottmann | 22 | 2000 | 4 |
| Hospital Clinico Universidad de Chile JJ Aguirre, Santiago | M Parra, S Quezada, L Quiroz | 4 | 2000 | 7 |
| ***Denmark*** |  |  |  |  |
| Aarhus University Hospital, Aarhus | L Hvidman, IM Mogensen, A Mouritzen | 51 | 5000 | 8 |
| Gentofte University Hospital, Hellerup | B Ostberg | 11 | 2000 | 4 |
| ***Egypt*** |  |  |  |  |
| Assiut University Hospital, Assiut | SNM Abdel-Samad, T Al-Hussaini, I El-Nashar | 22 | 8000 | 5 |
| ***Estonia*** |  |  |  |  |
| Tartu University Hospital-Women's Clinic, Tartu | F Kirss, K Rull, E Ustav, P Vaas | 31 | 2000 | 7 |
| ***Germany*** |  |  |  |  |
| CUB-Virchow Klinikum, Berlin | V Brink-Spalink, K Weizsaecker | 17 | 3500 | 8 |
| ***Hungary*** |  |  |  |  |
| University of Debrecen, Debrecen | T Major, R Poka | 1 | 2500 | 4 |
| ***Ireland*** |  |  |  |  |
| Coombe Women's Hospital, Dublin | S Daly | 35 | 8000 | 2 |
| ***Israel*** |  |  |  |  |
| Meir Medical Center, Kfar-Saba | H Kaneti, D Rosen, B Schachter | 34 | 5200 | 1 |
| Ma'ayney HaYeshua Medical Center, Bnei Brak | B Chayen, L Harel | 8 | 6600 | 7 |
| Edith Wolfson Medical Center, Holon | Z Hiaeb, G Malinger | 7 | 3000 | 3 |
| Soroka Medical Center, Beer Sheva | D Dukler, E Lunenfeld | 7 | 13500 | 9 |
| ***Jordan*** |  |  |  |  |
| Islamic Hospital, Amman | L AlFaris, M El-Zibdeh | 47 | 6000 | 3 |
| ***Poland*** |  |  |  |  |
| Medical University of Gdansk, Gdansk | I Domzalska-Popadiuk, P Kobiela, Z Pankrac, J Preis, K Preis, M Swiatkowska-Freund | 9 | 1800 | 6 |
| ***Portugal*** |  |  |  |  |
| Hospital Distrital de Faro, Faro | J Cravo | 3 | 2900 | 4 |
| ***South Africa*** |  |  |  |  |
| Stellenbosch University, Tygerberg | AM Theron, GB Theron | 27 | 7489 | 3 |
| University of Free State, Bloemfontein | HS Cronje, JM Plessis | 2 | 4000 | 7 |
| ***Spain*** |  |  |  |  |
| Hospital Clinic-University of Barcelona, Barcelona | M Munoz | 33 | 4105 | 3 |
| ***Sultanate of Oman*** |  |  |  |  |
| Khoula Hospital, Muscat | G Khan, S Khan | 31 | 4000 | 4 |
| ***The Netherlands*** |  |  |  |  |
| Atrium Medical Center, Heerlen | S Goossens, M Pieters, FJME Roumen | 59 | 1700 | 7 |
| Academisch Ziekenhuis Maastricht, Maastricht | F ten Cate, M Pieters, F Smits | 56 | 1200 | 10 |
| Sint Lucas Andreas Ziekenhuis, Amsterdam | M Heres, E Krabbendam | 28 | 1575 | 9 |
| ***United Kingdom*** |  |  |  |  |
| Bradford Royal Infirmary, Bradford | R Airey, D Farrar, DJ Tuffnell | 20 | 5500 | 1 |
| Royal Blackburn Hospital, Blackburn | V Heyes, C Melvin, C Schram | 18 | 3700 | 3 |
| Royal Hallamshire Hospital, Sheffield | A Galimberti, P Stewart | 9 | 7000 | 2 |
| Chesterfield Royal Hospital, Chesterfield | J Cresswell | 7 | 2860 | 1 |
| Nottingham City Hospital, Nottingham | C McCormick | 4 | 5700 | 3 |
| ***United States of America*** |  |  |  |  |
| University of Iowa Hospitals and Clinics, Iowa City | J Andrews, D Fleener | 16 | 1400 | 13 |
| Maricopa Medical Center, Phoenix | D Coonrod, BF Jimenez | 6 | 4000 | 11 |
| University of South Carolina, Columbia | S Brown, A Gregg | 5 | 2337 | 10 |
| New York University School of Medicine, New York | C Pitchford, D Seubert | 2 | 2000 | 12 |

*****Sites began recruitment to the trial at variable time points during the study.

**Experienced practitioners were those who are fully qualified obstetricians, midwives or family physicians who practice without supervision and judge themselves to be skilled at external cephalic version, with their Head of Department also judging them to be skilled at external cephalic version by providing signed documentation for the trial.

#### Appendix S2. Maternal mortality and morbidity

| Characteristic or outcome | | Early ECV (*n* = 765) ***n* (%)** | | Delayed ECV (*n* = 768) ***n* (%)** | |
| --- | --- | --- | --- | --- | --- |
|  | **Maternal death** | 0 | (0.0) | 0 | (0.0) |
|  |  |  |  |  |  |
|  | **Serious maternal morbidity during pregnancy, labour, birth or up to 28 days following birth** |  |  |  |  |
|  | Postpartum bleeding* | 15 | (2.0) | 12 | (1.6) |
|  | Documented blood loss of ≥ 1500 cm^3^ | 9 | (1.2) | 5 | (0.7) |
|  | Need for evacuation of the uterus with dilation/curettage and/or manual removal of the placenta after delivery | 8 | (1.1) | 7 | (0.9) |
|  | Laparotomy for haemorrhage | 2 | (0.3) | 0 | (0.0) |
|  | Blood transfusion required | 12 | (1.6) | 5 | (0.7) |
|  | Laparotomy (excluding caesarean section or tubal ligation) | 2 | (0.3) | 2 | (0.3) |
|  | Genital tract injury* | 13 | (1.7) | 6 | (0.8) |
|  | Hysterectomy | 1 | (0.1) | 0 | (0.0) |
|  | Vulvar or perineal haematoma requiring evacuation | 1 | (0.1) | 0 | (0.0) |
|  | Intraoperative damage to bladder, ureter or bowel requiring repair | 2 | (0.3) | 1 | (0.1) |
|  | Third or fourth degree tear involving the anal sphincter and/or mucosa | 9 | (1.2) | 5 | (0.7) |
|  | Thromboembolism requiring anticoagulant therapy | 0 | (0.0) | 1 | (0.1) |
|  | Deep vein thrombosis or thrombophlebitis | 0 | (0.0) | 1 | (0.1) |
|  | Systemic infection* | 4 | (0.5) | 6 | (0.8) |
|  | Maternal fever ≥ 38.5°C** | 4 | (0.5) | 4 | (0.5) |
|  | Pneumonia confirmed by X-ray | 0 | (0.0) | 2 | (0.3) |
|  | Sepsis confirmed by blood culture | 1 | (0.1) | 0 | (0.0) |
|  | Wound infection, dehiscence or breakdown* | 12 | (1.6) | 10 | (1.3) |
|  | Infection*** | 11 | (1.4) | 9 | (1.2) |
|  | Wound dehiscence or breakdown | 3 | (0.4) | 3 | (0.4) |
|  | Major medical life threatening illness | 2 | (0.3) | 2 | (0.3) |
|  | Disseminated intravascular coagulation | 2 | (0.3) | 1 | (0.1) |
|  | Bowel obstruction or paralytic ileus requiring nasogastric suctioning | 0 | (0.0) | 1 | (0.1) |
|  | Other serious maternal complication**** | 3 | (0.4) | 0 | (0.0) |
|  |  |  |  |  |  |
|  | **Maternal mortality or any serious maternal morbidity***** during pregnancy, labour, birth or up to 28 days following birth******** | 39 | (5.1) | 29 | (3.8) |
|  |  |  |  |  |  |
|  | **Other maternal morbidity** |  |  |  |  |
|  | Antibiotics after delivery before hospital discharge | 35 | (4.6) | 32 | (4.2) |

ECV, external cephalic version.

*More than one response may apply.

**On two occasions > 24 hours apart exclusive of first 24 hours postpartum.

***Resulting in prolongation of hospitalisation, readmission to hospital or repeated outpatient treatment.

****Other serious maternal complications in the early group included a bladder flap hematoma requiring rehospitalisation for bladder drainage, uterine rupture following vaginal birth after caesarean section and rupture of vulvar veins requiring embolisation.

*****There were no cases of fistula involving the genital tract, sympotomatic broad ligament haematoma, pulmonary embolism, adult respiratory distress syndrome, or amniotic fluid embolism.

******Relative risk = 1.35; 95% CI 0.84–2.16; *P* = 0.22.
